# Experimental Confirmation of a Whole Set of tRNA Molecules in Two Archaeal Species

**DOI:** 10.3390/ijms16012187

**Published:** 2015-01-20

**Authors:** Yoh-ichi Watanabe, Yutaka Kawarabayasi

**Affiliations:** 1 University of Tokyo, Graduate School of Medicine, Hongo 7-3-1, Bunkyo-ku, Tokyo 113-0033, Japan; E-Mail: ywatanab@m.u-tokyo.ac.jp; 2 Kyushu University, Faculty of Agriculture, Hakozaki 6-10-1, Fukuoka 812-8581, Japan; 3 National Institute of Advanced Industrial Science and Technology (AIST), Nakoji 3-11-46, Amagasaki, Hyogo 661-0974, Japan

**Keywords:** archaea, tRNA, tRNA gene, interrupted gene, genome, cDNA, intron, splicing, endonuclease

## Abstract

Based on the genomic sequences for most archaeal species, only one tRNA gene (isodecoder) is predicted for each triplet codon. This observation promotes analysis of a whole set of tRNA molecules and actual splicing patterns of interrupted tRNA in one organism. The entire genomic sequences of two Creanarchaeota, *Aeropyrum pernix* and *Sulfolobus tokodaii*, were determined approximately 15 years ago. In these genome datasets, 47 and 46 tRNA genes were detected, respectively. Among them, 14 and 24 genes, respectively, were predicted to be interrupted tRNA genes. To confirm the actual transcription of these predicted tRNA genes and identify the actual splicing patterns of the predicted interrupted tRNA molecules, RNA samples were prepared from each archaeal species and used to synthesize cDNA molecules with tRNA sequence-specific primers. Comparison of the nucleotide sequences of cDNA clones representing unspliced and spliced forms of interrupted tRNA molecules indicated that some introns were located at positions other than one base 3' from anticodon region and that bulge-helix-bulge structures were detected around the actual splicing sites in each interrupted tRNA molecule. Whole-set analyses of tRNA molecules revealed that the archaeal tRNA splicing mechanism may be essential for efficient splicing of all tRNAs produced from interrupted tRNA genes in these archaea.

## 1. Introduction

tRNA molecules carry amino acids that are attached to the 3' RNA terminus to ribosome for protein synthesis. Many tRNA molecules have been purified from original host organisms, and the respective tRNA nucleotide sequences were determined via direct RNA sequencing. Additionally, tRNA molecules are known to contain many different kinds of modified nucleotides.

Despite the long history of tRNA research, a whole set of tRNA molecules has been determined for only one organism—a bacterium *Mycoplasma carpicolum* [1]—because multiple tRNA genes have been detected for most triplet codons (isodecoders) in higher organisms. Before the era of genome sequencing, the actual number of tRNA genes that encode tRNA molecules with the same anticodon sequence was unknown. In higher organisms, therefore, splicing patterns were not experimentally determined because it was impossible to connect unspliced and spliced tRNA molecules to the respective tRNA gene from which they were transcribed. With the advent of whole-genome sequencing, it became evident that bacterial and eukaryotic organisms contained multiple isodecoders.

Archaea was differentiated from prokaryotic organisms approximately 30 years ago based on comparisons among 16S rRNA sequences [2]. Notably, many features of archaeal microorganisms (e.g., DNA replication, DNA repair, and regulation of transcription) were found to be more similar to those of the eukaryotic cells than those of the bacterial cells. Analyses of whole-genome sequence from multiple archaeal species indicated that only one tRNA gene was present for each triplet codon in most archaeal genomes. Therefore, we reasoned that analysis of tRNA complements solely from archaea species could provide important information on the actual transcription and actual splicing patterns of tRNA molecules. We attempted to obtain information on actual transcription of a whole set of tRNA molecules and the actual splicing patterns of interrupted primary tRNA transcripts in Crenarchaea because the complements of tRNA genes in Crenarchaeota contain larger numbers of interrupted tRNA gene than do those of Euryarchaeota species, which contain only 2–4 interrupted tRNA genes per genome.

In the splicing mechanism of interrupted tRNA molecules in Eukarya (at least for yeast and vertebrate), the mature tRNA structure, such as base-paring of the stem regions, is required for detection of the intron cleavage by their splicing machinery [3]. Conversely, in Archaea the final tRNA structure is not required for splicing; instead a bulge-helix-bulge (BHB) structure is the essential structure for correct splicing [4,5]. The typical BHB structure has a central 4-bp helix between the 5' exon and the intron region, flanking with 3' single-strand bulges in each strand and flanking helices between the 5' and 3' exons plus within intron [4,5].

In this chapter, we describe examples of experimental identification of whole sets of tRNA molecules from individual species, the respective splicing patterns, and the actual cleavage sites for splicing; these experiments involve synthesis of cDNAs from tRNA samples isolated from two Crenarchaeota species, *Aeropyrum pernix* and *Sulfolobus tokodaii*.

## 2. Confirmation of a Whole Set of tRNA Molecules

### 2.1. The Whole Set of tRNA Molecules in Aeropyrum pernix K1

#### 2.1.1. Introduction and Features of tRNAs in *A. pernix* K1

A hyperthermophilic archaeon, *Aeropyrum pernix*, was isolated in 1993 from a coastal sulfotaric thermal vent in Kodakara-jima Island in the Kyushu region of Japan [6]; this microorganism is aerobic and grows in the range of 90–98 °C with an optimal growth temperature of 95 °C. The entire nucleotide sequence of this Crenarchaeon was published in 1999 [7]. The entire genome sequence is 1,669,695 bp with approximately 56.3% G + C content, and approximately 2700 potential protein coding regions (open reading frames: ORFs), and one gene cluster encoding 16S and 23S rRNAs. Additionally, the computer software tRNAscan [8] predicted 44 tRNA genes including 11 interrupted tRNA genes, and three interrupted tRNA genes—tRNA^Asp^(GUC), tRNA^Thr^(UGU-1), and tRNA^Trp^(CCA)—were manually predicted from the genomic data. In the entire set of putative tRNA genes, two Thr(UGU) tRNA genes were predicted and tRNA gene with an A at the first position of anticodon region and a tRNA^Ile^(UAU) gene were not detected. Six tRNA genes were identified as three clusters consisting of two tRNA genes. Among the 14 interrupted tRNA genes, it was predicted that the tRNA genes encoding tRNA^Thr^(ACA) and tRNA^Trp^(UGG) were each interrupted by an intron at a position other than one base 3' from the anticodon region. To confirm the actuality of the putative tRNA genes and the introns within the interrupted genes, identification of the actual tRNA molecules transcribed from the predicted tRNA genes was attempted, as was confirmation of the splicing patterns of the interrupted tRNA genes containing the unusually long introns and the introns at unusual positions.

#### 2.1.2. Strategies for Detection of RNA Molecules

**Figure 1:**
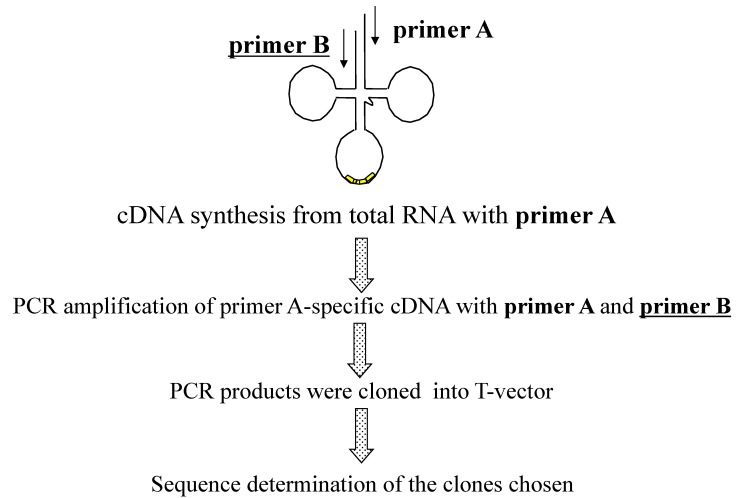
Overall strategy used to confirm predicted tRNA molecules in *A. pernix* K1.

The actuality of each putative tRNA molecule was confirmed based on sequencing of cDNA molecules synthesized from RNA molecule templates and the sequence-specific primer sets designed from the 5' and 3' end sequences of each putative tRNA gene [9]. The primers used for synthesis of the first strand of cDNA were designed from the 3' end sequences of each predicted tRNA gene. These primers and those designed from the 5' end sequences of each predicted tRNA gene were used for PCR amplification of cDNA molecules. Among the all tRNA genes detected, some tRNA genes share identical 3' end sequences and 5' end sequences. Ultimately, each of 21 different kinds of 18-mer 3' end-specific and 5' end-specific primers were designed [9]. These designed primers were used in different combinations to carry out 27 independent experiments for cDNA synthesis and subsequent amplification of cDNA. To maintain primer sequence specificity toward specific tRNA molecules, the solution containing the primers and RNA template was not cooled to temperatures below the annealing temperature. Each amplified cDNA fragment was cloned into a plasmid vector via the TA cloning method, and each cDNA fragment sequence was determined and analyzed [9]. The outline of the strategy used in this experiment is summarized in Figure 1.

#### 2.1.3. Isolation of cDNA of tRNA Molecules from Total RNA

**Figure 2:**
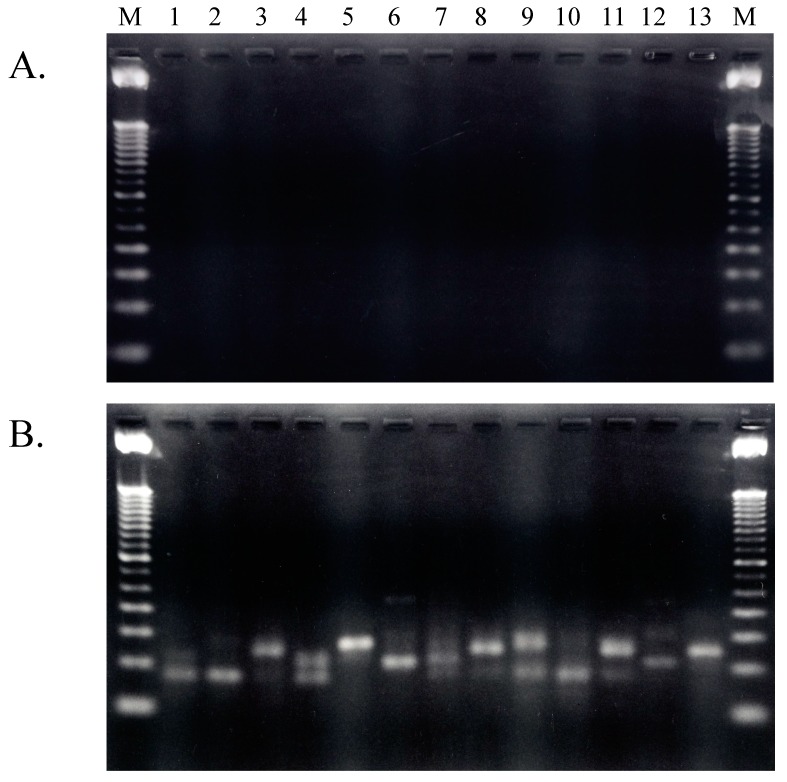
Agarose electrophoresis patterns of RT-PCR-amplified fragments. (**A**) Amplified without RT (reverse transcriptase); (**B**) Amplified with RT. Lanes 1 to 13 contain fragment that were amplified with primer sets 15–27 summarized in previous work; these primers were described previously [9]. M: 100 bp ladder marker.

Total RNA prepared from freshly cultured *A. pernix* cells was used as template for cDNA synthesis after removal of contaminating DNA via treatment with RNase-free DNase [9]. The quality of the total RNA preparation after removal of contaminating genomic DNA was checked by synthesis and amplification of cDNA in the absence of reverse transcriptase (RT). As shown in Figure 2, no PCR amplification was detected in experiments performed without RT; therefore, the purified total RNA molecules did not contain any contaminating genomic DNA and was successfully prepared by treatment with RNase-free DNase. Thus, this purified total DNA-free RNA was used for the first experiments involving cDNA synthesis of tRNA molecules present in living *A. pernix* cells.

Among the 27 primer sets used for RT-PCR amplification of cDNAs from the tRNA molecules in samples of total RNA, the primer set designed to amplify tRNA^Thr^(UGU-2) did not amplify any fragment. The fragments amplified with other 26 primer sets were cloned into plasmid vectors to construct libraries via TA-cloning technology. The nucleotide sequences of clones isolated from each cDNA library were determined.

Of the 22 uninterrupted tRNA molecules expected, transcription of 21 tRNA molecules were confirmed by sequencing of clones from the libraries constructed with total RNA samples and primer sets for only uninterrupted tRNA molecules. Although eight uninterrupted tRNA molecules were identified from six cDNA libraries constructed with primer sets for both uninterrupted and interrupted tRNA genes, three cDNA clones, one each for tRNA^Ser^(UGA), tRNA^Ser^(GGA), and tRNA^Phe^(GAA), were not identified. In all, 29 predicted uninterrupted tRNA molecules were confirmed by sequences of cDNA clones identified from the libraries that were constructed from total RNA samples.

For the predicted interrupted tRNA genes, identification of cDNAs representing the unspliced and spliced forms of each tRNA transcript was essential to determine the correct splicing patterns. From the libraries constructed from samples of total RNA and primer sets for interrupted tRNA genes, cDNAs were identified that represented an unspliced form of each predicted interrupted tRNA transcript, except for the tRNA^Thr^(CGU) transcript. Also, cDNA clones representing the spliced forms of each predicted interrupted tRNA transcript, except for the tRNA^Tyr^(GUA), tRNA^Ser^(CGA), and tRNA^Trp^(CCA) transcripts, were identified from libraries constructed with total RNA samples. A cDNA representing the spliced form of the tRNA^Thr^(CGU) transcript was identified from a total RNA library.

In all, four predicted uninterrupted tRNA molecules—tRNA^Phe^(GAA), tRNA^Ser^(GGA), tRNA^Ser^(UAG), and tRNA^Thr^(UGU-2)—and one unspliced and three spliced forms of cDNA for interrupted tRNA molecules were not identified from libraries constructed with the total RNA. To extend the search for cDNA clones representing these predicted, but as-yet-unconfirmed tRNA transcripts, additional libraries were constructed with small-sized RNA.

#### 2.1.4. Isolation of tRNA Molecules from Small-Sized RNA and Species-Specific Concentrated RNA

To isolate cDNA clones that were not identified in cDNA libraries constructed with total RNA, Qiagen RNA/DNA Kits (Hilden, Germany) were used to purify small-sized RNA samples that would be highly enriched with tRNA molecules. The appropriate primer sets for tRNA species not identified from total RNA and the small-sized RNA were used to synthesize and amplify cDNA molecules [9]; the amplified cDNA fragments were then cloned and used to construct cDNA libraries. From these libraries, cDNAs representing two putative uninterrupted tRNA molecules, tRNA^Ser^(GGA) and tRNA^Phe^(GAA), and the spliced form of tRNA^Tyr^(GUA) were isolated. Nevertheless, cDNA representing two predicted uninterrupted tRNA molecules and one predicted unspliced form and two spliced forms of predicted interrupted tRNA molecules were not recovered.

To further search for cDNA clones representing the five unidentified predicted tRNA molecules, the samples of small-sized RNA were further enriched for tRNA species via affinity chromatography. For this purpose, the tRNA sequence-specific DNA molecules were synthesized onto the 3' end of oligo(dT) that is covalently bound to magnetic beads, as shown in the previous report [9]. The tRNA species-specific concentrated RNA molecules were used to construct cDNA libraries for five predicted tRNA species. From these libraries, a cDNA clone representing only the spliced form of tRNA^Trp^(CCA) was recovered, and cDNAs representing each of the other four predicted tRNA species were not recovered. The findings regarding isolation of cDNAs representing each predicted tRNA molecule are summarized in Table 1. In all, 31 uninterrupted tRNA molecules and 12 spliced forms and 13 unspliced forms of interrupted tRNA molecules were confirmed as actual tRNA species based on isolation of cDNA molecules.

While an unspliced form of tRNA^Thr^(CGU) was not recovered from a cDNA, the actual splicing site was determined by comparing between genomic sequence and the sequence of the cDNA that represented the spliced form of tRNA^Thr^(CGU). Also tRNA^Ser^(UGA) and a spliced form of tRNA^Ser^(CGA) were not identified; therefore, more analyses are required to determine whether the gene predictions represent actual tRNA molecules. Conversely, an RT-PCR product representing tRNA^Thr^(UGU-2) was not recovered with any of three types of RNA template samples. These results indicate that the predicted tRNA^Thr^(UGU-2) gene was an overestimate and incorrectly identified as a gene; thus, tRNA^Thr^(UGU-2) gene may not be an actual gene.

**Table 1:** Summary of actual *A. pernix* K1 tRNAs confirmed by experimental identification of representative cDNAs [9].

tRNA Species	un	sp	tRNA Species	un	sp	tRNA Species	un	sp	tRNA Species	un	sp
Phe(AAA)	-	-	Ser(AGA)	-	-	Tyr(AUA)	-	-	Cys(ACA)	-	-
Phe(GAA)	-	s	Ser(GGA)	-	s	**Tyr(GUA)**	t	s	**Cys(GCA)**	t	t
Leu(UAA)	-	t	Ser(UGA)	-	x	End(UUA)	-	-	End(UCA)	-	-
Leu(CAA)	-	t	**Ser(CGA)**	t	x	End(CUA)	-	-	Trp(CCA) *	t	c
Leu(AAG)	-	-	Pro(AGG)	-	-	His(AUG)	-	-	Arg(ACG)	-	-
Leu(GAG)	-	s	Pro(GGG)	t	t	His(GUG)	-	t	Arg(GCG)	-	t
Leu(UAG)	-	t	Pro(UGG)	-	t	Gln(UUG)	-	t	Arg(UCG)	-	t
Leu(CAG)	-	t	**Pro(CGG)**	t	t	Gln(CUG)	-	t	Arg(CCG)	-	t
Ile(AAU)	-	-	Thr(AGU)	-	-	Asn(AUU)	-	-	Ser(AGU)	-	-
Ile(GAU)	-	t	Thr(GGU)	-	t	Asn(GUU)	-	t	Ser(GGU)	-	t
Ile(UAU)	-	-	**Thr(UGU) ^+^**	t, x	t, x	**Lys(UUU)**	t	t	**Arg(UCU)**	t	t
**Met(CAU) ^#^**	t	t	**Thr(CGU)**	x	t	**Lys(CUU)**	t	t	Arg(CCU)	-	t
Val(AAC)	-	-	Ala(AGC)	-	-	Asp(AUC)	-	-	Gly(ACC)	-	-
Val(GAC)	-	t	Ala(GGC)	-	t	Asp(GUC) *	t	t	Gly(GCC)	-	t
Val(UAC)	-	t	Ala(UGC)	-	t	Glu(UUC)	-	t	Gly(UCC)	-	t
Val(CAC)	-	t	Ala(CGC)	-	t	Glu(CUC)	-	t	Gly(UCC)	-	t

tRNA species are shown by amino acid species plus anticodon within parenthesis. tRNAs with introns are shown in bold and underlined; -, not predicted tRNAs; un, unspliced form; sp, spliced/uninterrupted form; t, identified from total RNA; s, identified from small RNA; c, identified from concentrated RNA; x, not identified; *, manually predicted tRNAs [7]; **^+^**, two tRNAs with UGU anticodon were predicted and the Thr(UGU-1) was predicted manually [7]; **^#^**, two tRNAs within three predicted tRNAs for Met were predicted as interrupted tRNAs.

#### 2.1.5. Confirmation of the Actual Splicing Patterns for Each Interrupted *A. pernix* tRNA Molecule

**Figure 3:**
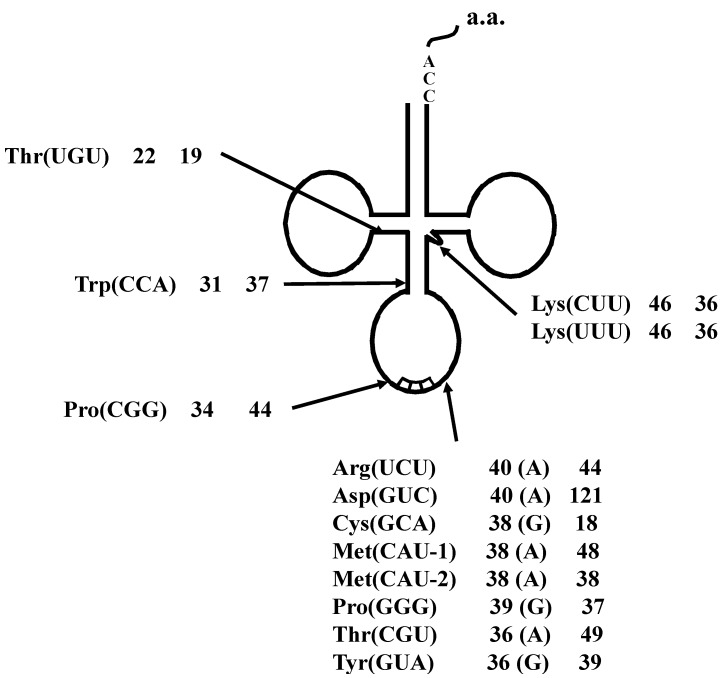
Summary of the tRNA introns identified in *A. pernix* K1. Each label includes the tRNA species, the respective intron positions from the 5' end of the respective tRNA, and the lengths of the introns. Characters within parentheses indicate the nucleotide one base 5' from the intron border.

By comparing between spliced and unspliced forms of each interrupted tRNA molecule, 13 actual splicing sites were determined. As shown in Figure 3, the introns of eight interrupted transcripts, including tRNA^Asp^(GUC) with its 121 bp-long intron, were confirmed to be one base 3' from the respective anticodon. The introns in the tRNA^Thr^(UGU) and tRNA^Trp^(CCA) molecules were confirmed to be 22 and 31 bases, respectively, from the 5' end of the respective tRNA molecule. Moreover, three additional introns, one each in tRNA^Pro^(CGG), tRNA^Lys^(CUU), and tRNA^Lys^(UUU), were identified at a location other than the conventional position.

**Figure 4:**
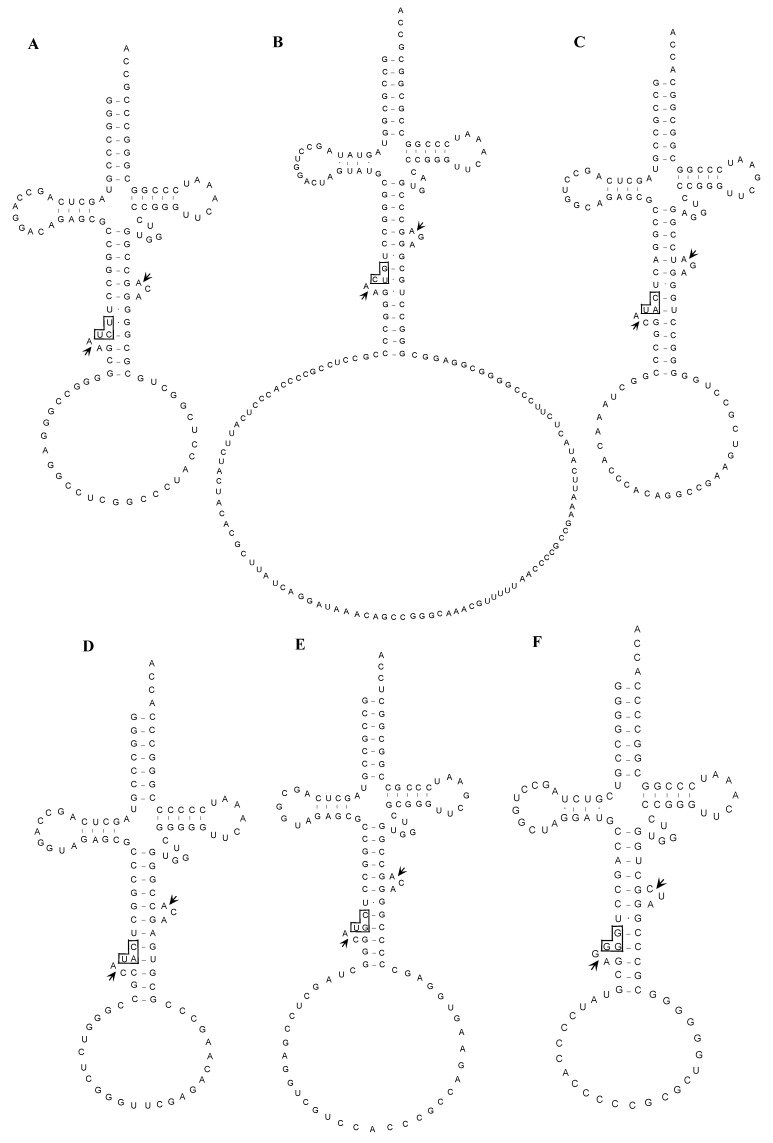

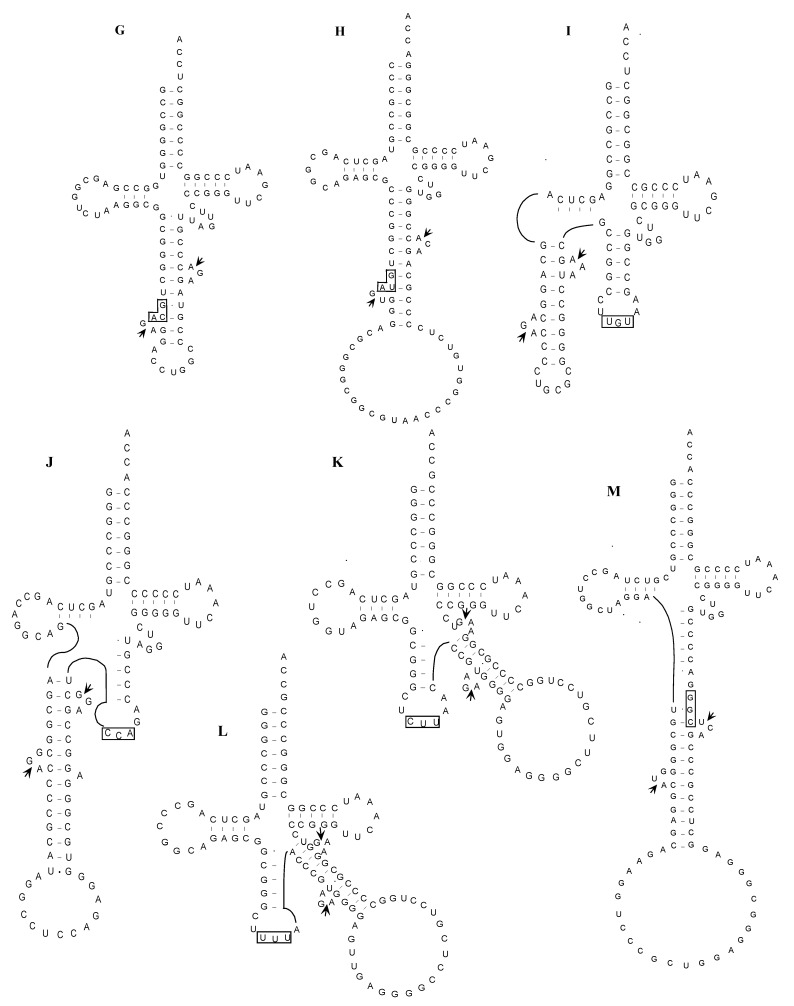
Proposed structures of the unspliced forms of interrupted tRNA molecules in *A. pernix* K1. Splicing sites and anticodon regions are indicated by short arrows and boxes, respectively. (**A**) tRNA^Arg^(UCU); (**B**) tRNA^Asp^(GUC); (**C**) tRNA^Met^(CAU-1); (**D**) tRNA^Met^(CAU-2); (**E**) tRNA^Thr^(CGU); (**F**) tRNA^Pro^(GGG); (**G**) tRNA^Cys^(GCA); (**H**) tRNA^Tyr^(GUA); (**I**) tRNA^Thr^(UGU-1); (**J**) tRNA^Trp^(CCA); (**K**) tRNA^Lys^(CUU); (**L**) tRNA^Lys^(UUU); (**M**) tRNA^Pro^(CGG).

Despite the position of the inserted intron, typical BHB structure was detected surrounding each actual splicing site as shown in Figure 4. This finding indicates that the BHB structure is essential for actual splicing of archaeal tRNA introns.

After publication of the *A. pernix* tRNA study [9], trans-splicing of *A. pernix* tRNA^Asp^(GCU) was reported [10]. In that report, transcription of the 3' half of the tRNA was driven from a putative promoter within the “intron” region of the tRNA gene. However, in the same study [10], the full-length unspliced tRNA molecule observed in the earlier study [9] was also detected; thus, *cis*-splicing of the unspliced tRNA could also occur in *A. pernix*.

### 2.2. The Whole Set of tRNA Molecules in Sulfolobus tokodaii *strain7*

#### 2.2.1. Introduction and Features of tRNAs in *S. tokodaii* strain7

**Figure 5:**
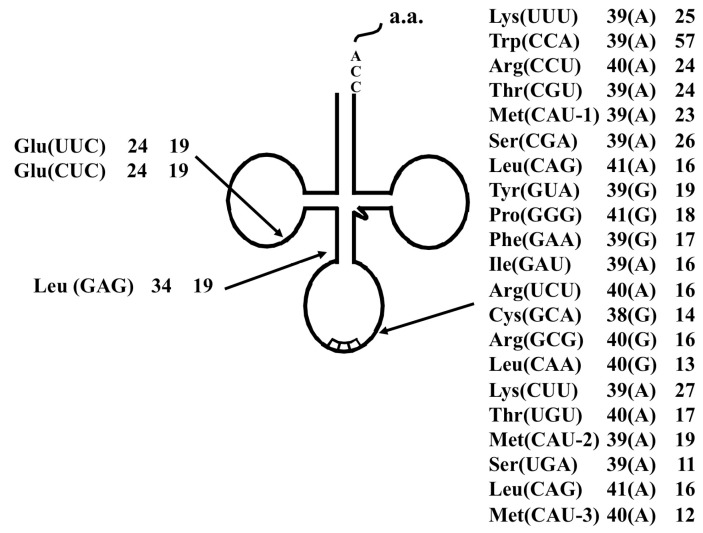
Summary of the tRNA introns identified in *S. tokodaii* strain7 [15]. Each label includes the tRNA species, the respective intron position relative to the 5' end of respective tRNA, and the lengths of the introns. Characters within parentheses indicate the nucleotide one base 5' from the intron border.

A thermoacidophilic archaeon, *S. tokodaii* strain7, was isolated from an acidic spa in Beppu Hot Springs in Kyushu, Japan. This microorganism is aerobic and grows optimally at 80 °C and pH 2.5–3 [11]. *S. tokodaii* strain7 belongs to the order Sulfoflobales, Crenarcaheota, and not the order Desulfolococcales, to which *A. pernix* K1 belongs [6]. The entire genomic sequence of this Crenarchaeon was published in 2001 [12]. The length of the genome is 2,694,756 bp with a G + C content of approximately 33%. This sequence is predicted to encode approximately 2800 ORFs and one rRNA gene cluster encoding 16S and 23S rRNAs. The computer software tRNAscan [8,13] and similarity search on this genome identified a complement of 46 predicted tRNA genes including 24 predicted interrupted tRNA genes, [12]. Therefore, *S. tokodaii* strain7 is apparently richer in tRNA introns than is *A. prenix* K1. A cluster of six predicted tRNA genes was identified. Of the 46 predicted tRNA genes, 44 lack a canonical 3' CCA sequence, which is usually present in bacterial and archaeal tRNA genes [14]. Among the 24 interrupted tRNA genes predicted, three (two tRNA^Lys^s and tRNA^Leu^(GAG)) are predicted to possess an intron in the D-arm and the anticodon stem, respectively, while other interrupted tRNA genes have the putative intron at position “37/38” (Table 2, Figure 5) [12].

**Table 2:** Summary of actual *S. tokodaii* strain7 tRNAs confirmed by experimental identification of representative cDNAs [14].

tRNA Species	un	sp	tRNA Species	un	sp	tRNA Species	un	sp	tRNA Species	un	sp
Phe(AAA)	-	-	Ser(AGA)	-	-	Tyr(AUA)	-	-	Cys(ACA)	-	-
**Phe(GAA)**	t	s	Ser(GGA)	-	HL	**Tyr(GUA)**	t	H	**Cys(GCA)**	t	s
Leu(UAA)	-	t	**Ser(UGA)**	t	HL	End(UUA)	-	-	End(UCA)	-	-
**Leu(CAA)**	t	t	**Ser(CGA)**	H	H	End(CUA)	-	-	**Trp(CCA)**	t	HL
Leu(AAG)	-	-	Pro(AGG)	-	-	His(AUG)	-	-	Arg(ACG)	-	-
**Leu(GAG)**	t	s	**Pro(GGG)**	t	HL	His(GUG)	-	t	**Arg(GCG)**	t	HL
**Leu(UAG)**	t	HL	Pro(UGG)	-	t	Gln(UUG)	-	t	Arg(UCG)	-	s
**Leu(CAG)**	t	HL	**Pro(CGG)**	t	t	Gln(CUG)	-	t	Arg(CCG)	-	t
Ile(AAU)	-	-	Thr(AGU)	-	-	Asn(AUU)	-	-	Ser(AGU)	-	-
**Ile(GAU)**	t	HL	Thr(GGU)	-	t	Asn(GUU)	-	t	Ser(GGU)	-	t
Ile(UAU)	-	-	**Thr(UGU)**	t	s	**Lys(UUU)**	HL	HL	**Arg(UCU)**	t	s
**Met(CAU) ^#^**	t	s	**Thr(CGU)**	t	s	**Lys(CUU)**	t	s	**Arg(CCU)**	t	HL
Val(AAC)	-	-	Ala(AGC)	-	-	Asp(AUC)	-	-	Gly(ACC)	-	-
Val(GAC)	-	tL	Ala(GGC)	-	s	Asp(GUC)	-	t	Gly(GCC)	-	t
Val(UAC)	-	t	Ala(UGC)	-	s	**Glu(UUC)**	t	HL	Gly(UCC)	-	t
Val(CAC)	-	t	Ala(CGC)	-	t	**Glu(CUC)**	HL	HL	Gly(UCC)	-	tL

tRNA species are shown by amino acid species plus anticodon within parenthesis. tRNAs with introns are shown in bold and underlined. -, not predicted tRNAs; un, unspliced form; sp, spliced/uninterrupted form; t, identified from total RNA; s, identified from small RNA; H, identified from total RNA treated with RNase H and oligo(s) targeted to specific tRNA(s); L, identified with longer primers; **^#^**, all of three predicted tRNAs with anticodon CAU were predicted as interrupted tRNAs [12].

#### 2.2.2. PCR-Based Identification of tRNA Molecules

**Figure 6:**
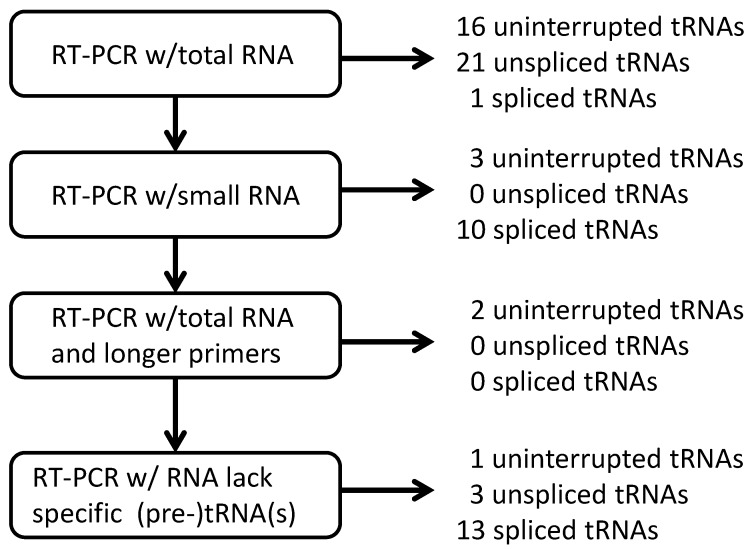
Overall strategy used to clone cDNAs that represent actual *S. tokodaii* tRNAs [15].

Samples of total or small-sized RNA were prepared from *S. tokodaii* cells according to the protocol described previously [9]. Initially, the protocol used in the *A. pernix* K1 tRNA study [9] was used to clone cDNAs representing each *S. tokodaii* strain7 tRNA (Figure 1), and cDNA clones representing 19 of the 22 predicted uninterrupted tRNA genes, 21 of 24 predicted unspliced tRNAs, and 11 of 24 spliced tRNAs were isolated (Figure 6, Table 2) [15]. These results indicate that detection of spliced tRNAs of *S. tokodaii* via this method is more difficult than detection of unspliced tRNAs. This result is surprising because spliced tRNA molecules should be more abundant than the corresponding unspliced tRNA based on a report by Hirata *et al.* [16]. More of the spliced tRNAs from *S. tokodaii* (13 out of 24) than from *A. pernix* K1 (four out of 14) were not detected by these protocols. Therefore, instead of enriching for the particular tRNA using sequence-specific affinity chromatography, as was done in the *A. pernix* tRNA study [9], we modified the protocol in two major ways: (1) we used longer primers (21–24-mer) for RT-PCR to improve the specificity and sensitivity of detection; and (2) we used targeted RNase H digestion (Figure 6) to eliminate the spliced/unspliced tRNA(s) that were possible competitors of our target tRNAs from the template RNA that was used for RT-PCR [15]. For example, for spliced tRNA^Lys^(UUU) detection, total RNA was treated with RNase H in the presence of an oligo DNA specific to intron sequence in the unspliced tRNA^Lys^(UUU) and the oligo DNA specific to tRNA^Lys^(CUU), because both unspliced tRNA^Lys^(UUU) and tRNA^Lys^(CUU) were possible competitors of spliced tRNA^Lys^(UUU) in RT-PCR. Using the RNase H-treated RNA as the template, a cDNA corresponding to the spliced tRNA^Lys^(UUU) was successfully amplified. Using a combination of these protocols, we ultimately cloned each of the predicted uninterrupted tRNAs, spliced tRNAs, and unspliced tRNAs (Figure 6, Table 2) [15].

#### 2.2.3. Identification of Cleavage Sites for Introns in tRNA Genes

**Figure 7:**
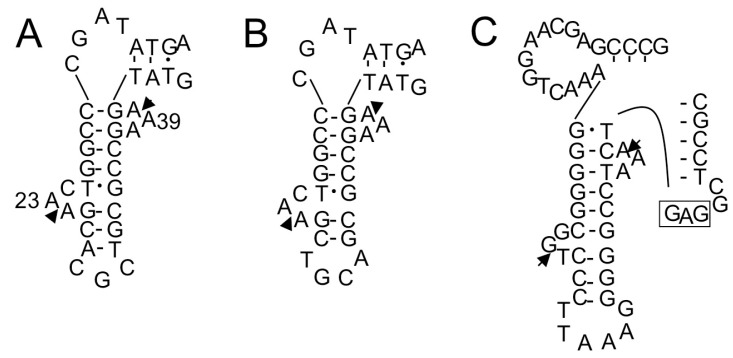
Exon-intron border regions of unspliced forms of tRNA^Glu^(UUC) (**A**); tRNA^Glu^(CUC) (**B**); and tRNA^Leu^(GAG) (**C**) of *S. tokodaii* strain7 [15]. These are examples of introns located at positions other than position “37/38”. In (**A**,**B**), only the D-arm region is shown; In (**C**), only the D-arm and anticodon arm regions are shown. The arrowheads indicate the exon-intron borders determined in our previous study [15]. The anticodon sequence of tRNA^Leu^(GAG) is boxed.

Based on comparisons between sequences representing spliced and unspliced transcript pairs from interrupted tRNA genes, we could not determine the precise exon-intron borders for 13 of 24 interrupted tRNA genes in *S. tokodaii* strain7 because of sequence redundancy at these borders [15]. For example, the nucleotide sequences of the 3' region of the 5' exon and the 5' region of the 3' exon of tRNA^Glu^(UUC) (Figure 7A) are C22-A23-A24-G25-C26 and a38-a39-a40-g41-t42. There are four possibilities for the spliced sequence: C22-a39-a40-g41-t42, C22-A23-a40-g41-t42, C22-A23-A24-g41-t42, and C22-A23-A24-G25-t42. Thus it was impossible to determine the precise exon-intron border based on the sequence of the corresponding cDNA (...CAAGT...). This limitation is shared with earlier genome-wide studies of tRNAs [9]. Although the prediction of a BHB structure may indicate the putative exon-intron borders, in some cases, typical BHB structures may not be present in these molecules. Instead, variations of BHB including mismatches in helices and/or bulges of with lengths other than three residues may be predicted. For example, the *A. pernix* unspliced tRNA^Lys^(CUU) does not form a helix that flanks the central helix between the 5' exon and the 3' exon (Figure 4K). The crenarchaeal splicing endonuclease (EndA) could cleave these varieties of BHB-like structures [17]. We have successfully expressed a functional, recombinant *S. tokodaii* EndA [18] and have used it to analyze the cleavage site of some synthetic unspliced *S. tokodaii* tRNAs *in vitro*. Consequently, we could determine the precise exon-intron borders of each interrupted tRNA [15].

**Figure 8:**
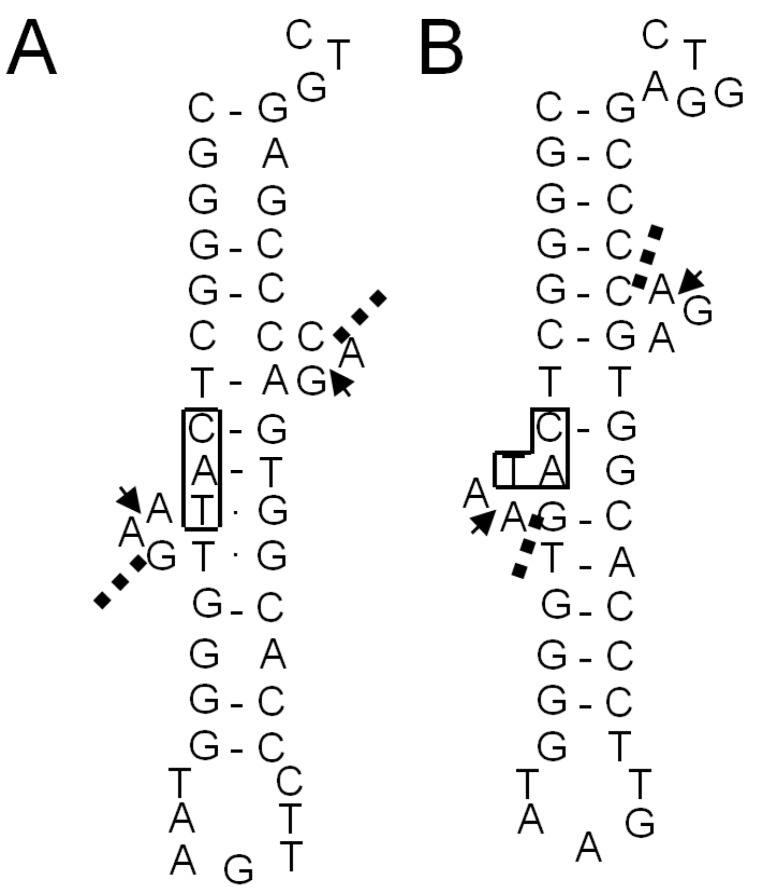
The BHB-like structure in the unspliced form of tRNA^Met^(CAU-1) of *S. tokodaii* strain7 [15]. Only the anticodon arm and extra loop regions are shown. (**A**) The structure predicted by Marck and Grosjean [19]; (**B**) the structure inferred by *in vitro* cleavage of the intron with recombinant *S. tokodaii* EndA [15]. The exon-intron borders determined by the biochemical study [15] are indicated by arrowheads. Dashed lines in (**A**,**B**) show the cleavage sites predicted by Marck and Grosjean [19]. The anticodon sequences are boxed.

The archaeal tRNA intron position places a BHB structure at each exon-intron border; the borders are located between the second and the third residues in the bulge loops that flank the central helix [17,19]. *In silico* prediction and identification of these BHB structure in tRNA genes have been performed [19,20]. However, such genomic studies (Figure 8A) [19] have not predicted the actual exon-intron borders in *S. tokodaii* strain7 tRNA^Met^(CAU-1) that were identified in our study involving recombinant *S. tokodaii* EndA (Figure 8B) [15]. When we compared our empirically determined structure with the prediction (Figure 8A) [19], the inferred BHB-like structure based on the established exon-intron borders (Figure 8B) have mismatches in the central helix of the BHB, but flanking helices have more base-pairs, suggesting that not only BHB core structure, but also the flanking region are important for positioning the cleavage sites. This result indicates that, in some cases, the experimental verification of tRNA expression and the intron position is necessary for determining tRNA gene transcription and intron location.

## 3. Conclusions

Many archaeal tRNAs have an intron at position 36/37 in the anticodon loop [17,19]. Of 13 interrupted *A. pernix* K1 tRNAs, eight have the intron at this position, and 21 of 24 interrupted *S. tokodaii* strain7 tRNA have the intron at this position (Figure 3 and Figure 5). However, recent genomic studies identified tRNA introns at position other than 36/37 (Figure 3 and Figure 5) [19,20]. In *A. pernix*, tRNA^Thr^(UGU-1) has an intron in the D-arm; tRNA^Trp^(CCA) has an intron in the anticodon stem; tRNA^Pro^(CGG) has an intron in the anticodon loop, but not at 36/37; and two tRNA^Lys^s have an intron in the variable arm (Figure 4). The predicted exon-intron borders in these tRNAs agreed with the cleavage sites indicated by the predicted BHB-like structure of the respective unspliced tRNAs [9]. In *S. tokodaii*, two tRNA^Glu^s have an intron in the D-arm, and tRNA^Leu^(CAG) has an intron in the anticodon stem (Figure 8). We also demonstrated that the actual cleavage of synthetic unspliced tRNA^Glu^s and tRNA^Leu^(CAG) of *S. tokodaii* by *S. tokodaii* EndA occurred as expected at the position of the predicted BHB-like structure (Figure 7A–C) [15]. Similar analyses involving a synthetic unspliced *A. pernix* tRNA^Thr^(UGU-1), and *A. pernix* EndA were also performed [16]. These are clear experimental demonstrations of cleavage of exon-intron borders at irregular positions in tRNAs by crenarchaeal EndAs.

By isolating cDNAs representing actual tRNA molecules and identifying the actual splicing events in the archaeal tRNA system, we have contributed important and useful information on expression and processing of tRNA genes.
